# To treat or not to treat tuberculosis –clinical decision making in patients with previous pulmonary tuberculosis using 18F-FDG PET/CT

**DOI:** 10.1016/j.rmcr.2023.101932

**Published:** 2023-10-19

**Authors:** Gunar Günther, Nebal Abu- Hussain, Peter M. Keller, Reto Guler, Sandra L. Mukasa, Karen Wolmarans, Friedrich Thienemann

**Affiliations:** a Department of Pulmonology and Allergology, Inselspital Bern, Bern University Hospital, University of Bern, Switzerland; b Department of Medical Sciences, School of Medicine, University of Namibia, Windhoek, Namibia; c Institute for Infectious Diseases, University of Bern, Bern, Switzerland; d International Centre for Genetic Engineering and Biotechnology (ICGEB), Cape Town Component, Cape Town, South Africa; e Institute of Infectious Diseases and Molecular Medicine (IDM), Department of Pathology, Division of Immunology, Faculty of Health Sciences, University of Cape Town, South Africa; f Wellcome Centre for Infectious Diseases Research in Africa, Institute of Infectious Disease and Molecular Medicine (IDM), Faculty of Health Sciences, University of Cape Town, South Africa; g General Medicine & Global Health (GMGH), Cape Heart Institute, Faculty of Health Science, University of Cape Town, South Africa; h Department of Medicine, Faculty of Health Science, University of Cape Town, South Africa; i Department of Internal Medicine, University of Zürich, Switzerland

**Keywords:** recurrent, Tuberculosis, 18F-FDG PET/CT, False-positive

## Abstract

Post-tuberculosis (TB) radiological changes and symptoms can mimic TB. PCR-based diagnostic tests can show positive results, suggesting the presence of *Mycobacterium tuberculosis* DNA in the absence of viable bacteria. We present a case with two episodes of previous TB. Despite workup including trace to low positive PCR results, after performing sputum analysis, bronchoalveolar lavage analysis, cyto-brush and 18F-FDG PET/CT guided transthoracic biopsy, no culturable mycobacteria were detected. 18F-FDG PET/CT showed a high metabolic activity of the biopsied lesions. More accurate strategies and tools in patients with previous TB and positive PCR results are required to make appropriate treatment decisions.

## 1 Introduction

Tuberculosis (TB) remains a global threat with an estimated 9.9 million new cases in 2021 of which only 5.8 million patients were diagnosed with TB [1]. While many factors contribute to these missed cases, the optimal diagnostic test and management of patients with recurrent TB symptoms and persistent radiological lesions on chest X-ray (CXR) or computed tomography (CT) remains a clinical challenge. Modern *Mycobacterium tuberculosis* (*Mtb.*) DNA based molecular diagnostics have improved the sensitivity of TB diagnostics, but due to complex host-pathogen interactions of TB, the presence of *Mtb.* DNA in a diagnostic specimen, especially in patients with previous history of TB does not by itself justify initiation of TB treatment [[2], [3], [4]]. TB cultures are still considered the reference standard for diagnosing viable *Mtb*. It will only yield results after multiple days and only report “no growth” after 8 - 12 weeks of incubation and long uncertainty for patients and physicians. Therefore, many clinicians, equipped with limited diagnostics tools, frequently have to make challenging clinical decisions, especially in patients with previous TB disease, residual radiological lesions and new onset or worsening respiratory symptoms compatible with TB.

We herewith present a clinical case where we used the entire spectrum of diagnostic tools available in an high-income setting and thus not available to the large majority of patients at risk for TB to decide about optimal management. However diagnostic uncertainty remained.

## 2 Case presentation

A 33-year-old male migrant from the Gambia presented in 2019 with epigastric pain, a history of weight loss and fatigue to the University Hospital of Bern, Switzerland. He denied fever, cough, and night sweats. There was no nausea, vomiting or diarrhoea reported. In the medical history, he reported that he was treated for pulmonary TB twice in the Gambia, approximately 5 and 10 years ago for 6 and 8 months, respectively.

**Fig. 1:**
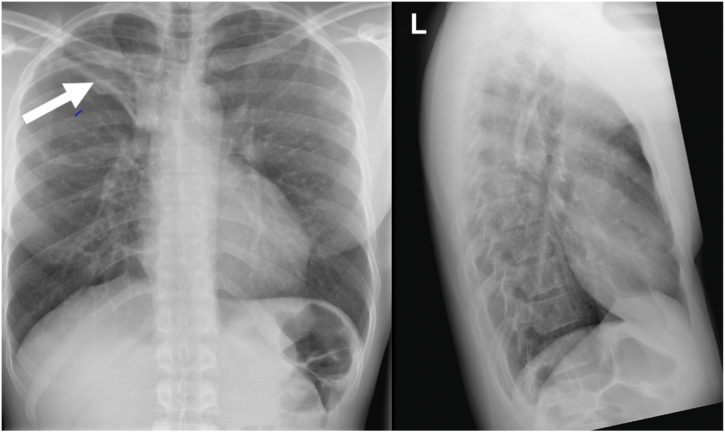
Chest X-ray (CXR) of a 33-year-old male from the Gambia with previous history of tuberculosis who presented with epigastric pain, weight loss and fatigue. The CXR shows consolidation in the upper zone of the right lung (white arrow).

The vital parameters and the clinical exam were unremarkable. Full blood count, electrolytes, creatinine, and liver function tests were normal. HIV serology was negative, as was a *Helicobacter pylori* antigen test of the stool. Further workup followed with a CXR and an abdominal ultrasound. While the ultrasound showed no abnormality, the CXR revealed consolidation in the upper zone of the right lung and was described as suspicious for pulmonary TB by the local radiologist (Fig. 1).

**Fig. 2:**
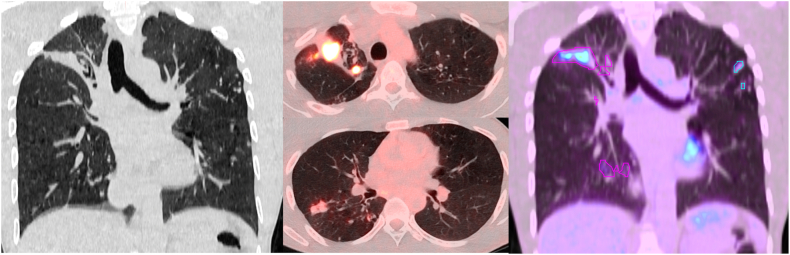
Computed tomography (CT) and 18F-FDG-PET/CT scans of a 33-year-old male from the Gambia with previous history of tuberculosis who presented with epigastric pain, weight loss and fatigue. The CT scan revealed a consolidation in the right upper lobe consistent with post-tuberculosis lung disease such as fibrotic bands (left image, coronal CT scan). The PET/CT shows FDG-avid hot lesions (SUVmax 9.06 SUVbw) in the right upper lobe (top middle picture) and a smaller consolidation in the middle lobe (SUVmax 2.69 SUVbw, bottom middle picture). Total lung glycolysis (TLG) is a semiautomated technique to calculate the total lung glycolytic activity above a threshold (background lung avidity above SUVmax = 1, right picture). Highlighted regions of interest (ROIs) are circulated in pink and light blue. (For interpretation of the references to colour in this figure legend, the reader is referred to the Web version of this article.)

A CT chest demonstrated a dense nodular consolidation in the apex of the right lung and a small cavitary lesion with discrete tree-in-bud and ground glass opacification surrounding the cavity (Fig. 2). The CT scan findings supported the suspected recurrent episode of TB.

Further workup for active pulmonary TB was initiated. Smear microscopy and polymerase chain reaction (PCR) diagnostics (Xpert MTB/RIF Ultra, Cepheid, Sunnyvale, CA, US) from sputum samples were negative. Subsequently, a bronchoalveolar lavage (BAL) was performed, which detected *Mtb*. DNA with a “trace” on quantification. Resistance to rifampicin was reported as “indeterminate” due to low quantity of *Mtb*. DNA. The result of the BAL prompted us to repeat an analysis from sputum on the next day, which was now reported as *Mtb*. DNA “very low”, and rifampicin resistance was detected. Due to the clinical implications of this result, a second BAL was performed. PCR results revealed *Mtb.* DNA “trace”, rifampicin resistance “indeterminate” and endobronchial brushing yielded *Mtb*. DNA “low”, and no rifampicin resistance was detected (Table 1).

**Table 1:** Results of diagnostics specimen (smear microscopy, Xpert MTB/RIF Ultra for tuberculosis detection and rifampicin resistance, *Mycobacterium tuberculosis* culture) in a 33-year-old male from the Gambia (Abbreviation: BAL, bronchoalveolar lavage).

Date	Specimen	Smear	PCR Xpert MTB/RIF Ultra	Xpert Rifampicin resistance	Culture
17.-19.04.2019	3 x Sputum	negative	negative	not possible	Negative (12 weeks)
23.04.2019	BAL	negative	trace	indeterminate	Negative (8 weeks)
24.04.2019	Sputum	negative	very low	detected	Negative (12 weeks)
25.04.2019	Sputum, induced	negative	trace	indeterminate	Negative (12 weeks)
26.04.2019	BAL	negative	trace	indeterminate	Negative (8 weeks)
26.04.2019	Brush	negative	low	not detected	Negative (12 weeks)
07.05.2019	CT biopsy	negative	negative	not possible	Negative (12 weeks)

This complex diagnostic scenario allowed the following conclusion: in a patient with two previous episodes of TB disease and new onset of TB symptoms (weight loss and fatigue) a minimal amount of *Mtb*. DNA was detectable, potentially with the presence of mutations, conferring resistance to rifampicin. The options to initiate treatment for drug-resistant TB or to await *Mtb.* cultures were discussed. Nevertheless, considering the social circumstances of the patient and the challenges of adherence and management of side effect of drug-resistant TB treatment and the negligible risk of contagiousness with only “trace” or “very low” quantity *Mtb.* DNA on PCR test, we opted for a further diagnostic workup to understand the metabolic activity of the detected lung lesions.

Based on the inflammatory characteristics of active TB disease, a [^18^F]2-fluoro-2-deoxy-d-glucose (FDG) positron emission tomography CT scan (PET/CT) was performed to evaluate the metabolic activity of the pulmonary lesions. The PET/CT demonstrated high FDG uptake (SUVmax 9.06 SUVbw, total lung glycolysis (TLG) of 151.62 SUVbw*ml) with a total lesion volume above SUV = 1 of 63.0 ml of the apical lesion of the right lung, suggesting a highly inflamed and active process as opposed to cold lesions that have been described as scarred tissue of previous TB (Fig. 2). The decision was taken to perform a CT-guided biopsy of the lesion with the highest FDG uptake: despite demonstrating granulomatous tissue Xpert MTB/RIF Ultra and smear microscopy of the tissue sample remained negative.

Those results led to the decision, to not start treatment for drug resistant TB (MDR-TB), but to await TB culture results (Bactec MGIT, Becton Dickenson, Franklin Lakes, NJ, US). The patient was subsequently discharged from our hospital, his abdominal symptoms had subsided, and no other reason for the weight loss and fatigue could be established. TB cultures from all collected sputum, BAL brushing, and biopsy specimen remained negative after prolonged incubation (12 weeks, Table 1). Unfortunately, the patient could not be traced for follow-up.

## 3 Discussion

We present a case with two previous episodes of TB treatment, new onset of non-specific TB symptoms, residual lung inflammation on PET/CT, identification of *Mtb.* DNA, but no culturable and thus viable *Mtb*. bacteria on sputum, BAL and tissue samples. The case illustrates the paradigm of TB as a continuous spectrum of disease and challenges of correct interpretation of test results, despite the employment of very advanced diagnostic methods.

Xpert MTB/RIF is one of the most established molecular tests for *Mtb*. detection and determination of rifampicin resistance. Its new version Xpert MTB/RIF Ultra shows increased sensitivity (pulmonary TB: 90.9 % versus 84.7 %, using positive culture as gold standard) and reports a “trace” result of *Mtb*. DNA, compared with its previous version. This comes at a price of slightly reduced specificity (95.6 % versus 98.4 %) [5,6]. The interpretation of “trace” results remains challenging considering the high sensitivity of the Xpert MTB/RIF Ultra test [2,7]. Importantly, the test cannot differentiate dead and viable *Mtb*. bacteria. In patients with previous TB - as presented here - “trace” results (and here even “very low”) remained culture negative in sputum, BAL and tissue biopsy, suggesting the absence of viable *Mtb*. in the pulmonary lesions. WHO guidelines suggest that children, patients living with HIV, extrapulmonary TB and patients with no history of TB or TB treatment in the past five years should be treated with a first-line TB treatment regimen following a positive Xpert MTB/RIF Ultra “trace” result, while those with previous TB or treatment during the last five years should undergo clinical re-evaluation, employment of other tests (including TB culture) and treatment initiation based on clinical judgement [8]. In this case, considering the discrepancy of Xpert MTB/RIF Ultra results, applying meaningful clinical judgement to initiate treatment remained challenging.

**Fig. 3:**
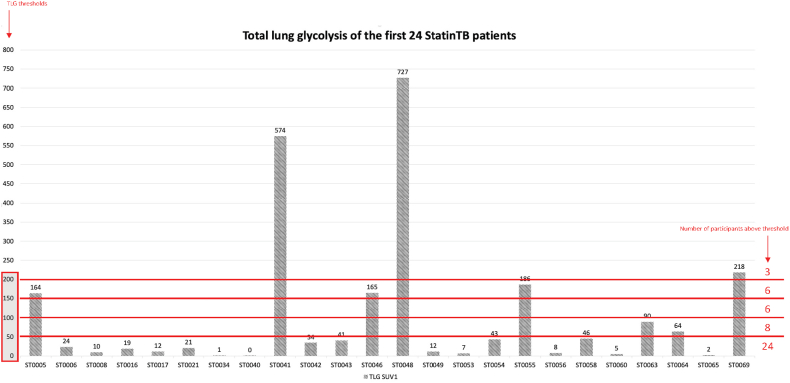
Total lung glycolysis (TLG) of the first 24 participant enrolled into the StatinTB trial. Six study participants had a TLG above 150 SUVbw*ml. Our patient reported here had a TLG of 151.62 SUVbw*ml.

We therefore performed a PET/CT scan to investigate the metabolic activity of the lesions with the aim to differentiate between old post infective scarring, often referred to as post-tuberculosis lung disease (PTLD) [9] with fibrotic bands, fibro-cavitation, bullae and pulmonary nodules or an active infective lesion with high metabolic activity. In our patient the lung lesions had high uptake of FDG. These findings are in line with reports from South Africa. Malherbe et al. described a high prevalence of active pulmonary lesions on PET/CT in patients who recently completed TB treatment and were discharged from the TB control program [10]. Those patients did not grow *Mtb*. in culture, but *Mtb*.-specific RNA and *Mtb*. DNA in BAL samples could be detected suggesting the presence of viable *Mtb*. and a relevant role of immunological responses to controlling TB after successful treatment and preventing relapse. We further assessed total lung glycolysis (TLG) as a marker of metabolic activity of both lung volumes as described in the PredictTB protocol [11]. We compared TLG of this patient with a cohort of 24 patients, who recently completed TB treatment, as part of the StatinTB trial currently conducted by our group at the University of Cape Town (ClinicalTrials.gov Identifier: NCT04147286). Our patient's TLG of 151.62 SUVbw*ml was as high as the 6/24 StatinTB participants (above 150 SUVbw*ml, Fig. 3) with the highest TLG. All 24 study participants were sputum and culture negative for *Mtb.*, as well as Xpert MTB/RIF Ultra negative at the time TB treatment completion and PET/CT acquisition. One of the 24 (4.2 %) StatinTB trial participants did relapse during the following six months of observation. Hence, a high FDG uptake does not necessary correlate with the presence of viable *Mtb*. The decision not to treat the patient, especially in the context of the possible presence of rifampicin resistance was taken, also considering PET/CT findings, using all available diagnostic tools in our setting and weighing carefully risks and benefit.

## 4 Conclusion

• The diagnosis of recurrent TB and appropriate treatment decision making remains an extremely challenging task for clinicians independent of the diagnostic tools available.
• It remains unclear if detectable *Mtb.* DNA and high metabolic activity on PET/CT in the absence of a positive *Mtb.* culture growth can predict TB relapse.
• More research is needed and under way to better understand persistent inflammation at the end of TB treatment and to optimise TB treatment to prevent relapse and post-tuberculosis lung disease.

## Funding source

This publication was produced by StatinTB consortium which is part of the EDCTP2 programme supported by the 10.13039/501100000780European Union (grant number RIA2017T-2004-StatinTB).

## Author contribution

GG, NAH and FT drafted the manuscript, PMK, KW and RG revised the manuscript; KW, RG and FT collected data, all authors approved the final version.

## Declaration of competing interest

No conflict of interest
